# Bi-directional associations of core affect and physical activity in adults with higher body weight: An ecological momentary assessment study

**DOI:** 10.1177/13591053241228202

**Published:** 2024-01-29

**Authors:** Caroline Seiferth, Janis Fiedler, Tanja Färber, Magdalena Pape, Stefanie Schroeder, Stephan Herpertz, Sabine Steins-Loeber, Jörg Wolstein

**Affiliations:** 1 University of Bamberg, Germany; 2 Karlsruhe Institute of Technology (KIT), Germany; 3 LWL-University Hospital, Ruhr University Bochum, Germany

**Keywords:** accelerometry, affective states, ambulatory assessment, vector magnitude, obesity

## Abstract

Affect is known to be predictive of and enhanced by higher physical activity (PA) levels in the general population. This secondary analysis aimed to increase the understanding of the bi-directional relationship between PA and core affect (i.e. valence, energetic arousal, and calmness) among adults with higher body weight. Affect and PA were assessed in naturalistic settings via ecological momentary assessment using a mixed sampling scheme from 157 participants (body mass index: 32.99 ± 3.78 kg/m^2^). Multilevel models revealed that being more physically active in the 15 minutes prior to the assessment predicted an increase in energetic arousal and a decrease in calmness. Subsequently, feeling more energetic and agitated was associated with increased PA within the following 15 minutes. Valence (i.e. pleasure–displeasure) was not associated with PA nor predictive of subsequent PA. Digital PA interventions may target the enhancement of feelings of energy and present psychoeducation about these distinct psychological benefits.

## Background

Being physically active on a regular basis in everyday life is linked to physical and mental health benefits for individuals with higher body-weight (Carraça et al., 2021; Oppert et al., 2023). However, research suggests that fewer higher-weight individuals are likely to achieve sufficient physical activity (PA) levels compared to “normal-weight” counterparts (Cassidy et al., 2017; Ladwig et al., 2021). Hence recognizing dynamic real-life facilitators or barriers to increase PA in everyday life is important for identifying targets for PA promotion in obesity management.

Affect has been highlighted as a meaningful predictor and outcome of PA in the general population (Bryan et al., 2017; Conroy and Berry, 2017; Ekkekakis and Brand, 2019; Forster et al., 2021; Reed and Ones, 2006; Stevens et al., 2020; Williams and Evans, 2014). The hedonic theory proposes that a positive affective response (i.e. feeling good) during or following PA is associated with a higher likelihood to repeat the behavior and thus engage in more structured (Bryan et al., 2017; Conroy and Berry, 2017; Emerson and Williams, 2015; Lyubomirsky et al., 2005; Rhodes and Kates, 2015) and incidental activity (e.g. walking, cleaning; Le et al., 2022). Additionally, increased PA can lead to positive affective outcomes indicating a dynamic bidirectional relationship (Emerson and Williams, 2015; Hyde et al., 2011) which has been replicated at different time intervals in intensive longitudinal studies in healthy children, adolescents, and adults (e.g. Bourke et al., 2021; Kim et al., 2020; Liao et al., 2015, 2017; Ruissen et al., 2022).

Affect can be conceptualized as core affect, which is defined as a “neurophysical state consciously accessible as a simplest raw (nonreflective) feeling evident in moods and emotions” (Russell, 2003: 148). Across the literature, there is an ongoing discourse about how to conceptualize and measure core affect (Ekkekakis, 2013; Williams et al., 2019). Based on the circumplex model of core affect (Russell, 1980), affect can be summarized along two independent dimensions of valence (i.e. pleasure–displeasure) and arousal (i.e. activation–deactivation). Thayer (1990) rejected valence as a separate dimension and claimed that two distinct types of arousal exist: energetic arousal (i.e. tiredness–wakefulness) and tense arousal (i.e. relaxation–tension). In contrast, the two-dimensional approach from Watson and Tellegen (1985) described affect along the dimensions of positive affect (e.g. happiness, excitement, alertness) and negative affect (e.g. distress, anger, fear). A three-dimensional model based on valence, energetic arousal, and calmness (Schimmack and Grob, 2000; Schimmack and Rainer, 2002) has been proven to be sensitive enough to measure fluctuations of core affect within-person in daily life (Wilhelm and Schoebi, 2007) and is used in this paper.

Existing studies that investigate the dynamic relation between dimensions of core affect and incidental PA in everyday life usually use ecological momentary assessment (EMA). EMA allows to assess near real-time data and thus limit retrospective bias, improving ecological validity by assessing the data in everyday life, and to disentangle within- (i.e. how a person’s associations between core affect and PA change within this person over minutes, days, or weeks) and between-person (i.e. how associations differ between persons with higher average core affect/PA from persons with lower average core affect/PA) associations (Kanning et al., 2013; Liao et al., 2016). Such intensive longitudinal assessments allow measuring detailed and temporal variations between affect and PA, which are possible reasons for physical inactivity in everyday life.

Despite the benefits mentioned above, most of the studies investigating the relationship between core affect and PA in higher body-weight individuals to date have been conducted in laboratory settings and focus on structured PA sessions and between-person effects (e.g. Berger et al., 2023; Ekkekakis et al., 2010; Ekkekakis and Lind, 2006; Hulens et al., 2003; Unick et al., 2012, 2015). Moreover, a range of negative emotional experiences that may influence the relationship between affect and PA in individuals with higher body weight have been identified (Baillot et al., 2021; Hamer et al., 2021; Thedinga et al., 2021). The combination of psychological (e.g. fear of embarrassment and weight discrimination, internalized weight stigma, lower self-efficacy) and physical (e.g. discomfort, perceived pain) distress may be a determinant for decreased PA engagement in this group (Ekkekakis et al., 2016).

However, these results provide little information about how affect and PA are related in real-life among adults with higher body-weight. To deepen this understanding, EMA studies are needed that examine the nature and frequency of time-varying within-person associations between core affect and device-based PA (Engel et al., 2016). Previous EMA studies have investigated this reciprocal relationship at the day-level and found significant within-person associations between PA and valence (Carels et al., 2007; Emerson et al., 2018) and negative affect (Kerrigan et al., 2020).

Although the understanding of the temporal dynamics of bidirectional within-person associations is inconclusive (Kim et al., 2020), core affect is thought to change rapidly within-persons (Brose et al., 2020) and that the association between affect and PA is predicted to be greatest during or immediately after PA (Reichert et al., 2017). It is therefore essential to investigate the magnitude of the effect within shorter timeframes to gain a deeper understanding about the timing of the affect-PA relation in a sample of higher-weight individuals. Such momentary associations have been primarily evaluated in the general population analyzing short time windows of PA in the 10 or 15 minutes before and following an EMA (Bossmann et al., 2013; Kanning et al., 2015; Liao et al., 2017; Reichert et al., 2017). More PA was associated with higher valence (Bossmann et al., 2013; Liao et al., 2017; Schwerdtfeger et al., 2010) and higher energetic arousal (Bossmann et al., 2013; Kanning et al., 2015; Liao et al., 2017; Reichert et al., 2017) and vice versa (Kanning and Schoebi, 2016; Koch et al., 2018; Liao et al., 2017; Reichert et al., 2016). Concerning calmness, bidirectional associations between higher PA and lower calmness were identified (Kanning et al., 2015; Koch et al., 2018; Liao et al., 2017; Reichert et al., 2016, 2017).

Building on the above findings, the current EMA study aimed to investigate the bi-directional association between estimates of PA and dimensions of core affect (i.e. valence, energetic arousal, and calmness) in a sample of higher-weight individuals at the within- and between level using accelerometer-derived PA counts continuously measured in everyday life over seven consecutive days. The first objective of this secondary analysis was to examine whether PA in the *15 minutes prior* to the EMA is associated with participants reports of core affect at the within-person levels. We hypothesized that higher levels of PA *15 minutes prior* to an affect measurement occasion would be associated with higher reported levels of valence (H1.1) as well as energetic arousal (H1.2) and lower reported levels of calmness (H1.3). The second objective was to investigate whether participants’ reports of core affect would be associated with PA in the *following 15 minutes* at the within-person levels. We hypothesized that higher levels of valence (H2.1) and energetic arousal (H2.2) and lower levels of calmness (H2.3) at one EMA would be associated with higher levels of PA in the *following 15 minutes*. We further explored differences at the between-person level and controlled for both person-specific (sex, age, BMI) and study-related variables (day of the week, time of the day, day in the study).

## Methods

This is a pre-registered secondary analysis of existing EMA data from a randomized controlled trial (RCT) that tested the effectiveness of the gender-sensitive mHealth intervention I-GENDO (Pape et al., 2022; Seiferth et al., 2023).

### Participants and procedure

Participants were recruited via social media, newspaper, radio, and self-help groups from December 2019 to August 2020. Eligible individuals (Additional File 1) initially attended an in-person appointment at the study sites to receive an accelerometer and the login information for the I-GENDO application. The design of the original RCT was that participants completed a 7-day assessment (data collection: December 2019 to August 2020), followed by a 12-week intervention period and a second 7-day assessment. The follow-up assessments took place 9 and 15 months after study inclusion. Participants were informed of their group allocation after the first 7-day assessment was completed. For the current examination, only data from those participants who participated in the first assessment and who both wore the accelerometer and answered at least one EMA questionnaire were included (*n* = 196, 68% female). Participants received a monetary compensation of 10€ for each day with at least 10 hours accelerometer wear-time and 30€ for completing the first questionnaire of the RCT.

### EMA data collection procedure

A mixed sampling scheme with both semi-random signals and participant-initiated prompts was applied. Participants chose their preferred time frame of assessment (e.g. 7 a.m.–10:30 p.m), which could be adapted during the 7-day assessment. Participants were alerted by the app at random times within eight 90-minute blocks that were 30 minutes apart throughout the chosen time period.

Participants were instructed to respond to the prompt as soon as possible in everyday life. EMA questionnaires consisted of 18 items in total with an estimated response time between 30 and 120 seconds. Only the items measuring core affect were included in this examination. For these items, participants marked the point that represented their perception of their current state on a visual analog scale. If participants missed a prompt or were not able to answer the questions at the moment, they were able to postpone the questions. Participants were not aware of the prompting schedule and were only informed that prompts would occur randomly in the chosen time span. Participants’ compliance with the sampling schedule was calculated from the completed number of assessments relative to the 56 scheduled assessments.

### Measurements

Questions about age, sex, height, and weight were included in the questionnaire of the RCT. BMI was calculated by dividing the reported weight in kilograms by height in meters squared.

#### Device-based measured PA

PA in everyday life was measured continuously using the tri-axial ActiGraph^®^ wGT3X-BT accelerometer (firmware v1.9.2, ActiGraph, Pensacola, FL, USA). The accelerometers have shown good validity and reliability in adults in previous studies (Aadland and Ylvisåker, 2015). Participants were instructed to position the sensor at the right hip, which was found to be a good placement for the assessment of everyday PA (Cleland et al., 2013) and to wear the accelerometer during waking hours and to only take it off while showering or participating in other water-related activities. PA as measured by the accelerometer was expressed as vector magnitude (VM) of 60-second epochs, which combines the mean acceleration from three individual axes $\left(vm=\sqrt{\left(axis\;1^{2}\right)+\left(axis\;2^{2}\right)+\left(axis\;3^{2}\right)}\right)$ . VM was choosen over certain intensity estimations like moderate-to-vigorous PA for better comparability throughout studies as for example, the choice of certain cut-points varies greatly throughout the literature (Burchartz et al., 2020). VM represents a dimensional estimate for PA over a defined time period (Migueles et al., 2022) and is used for estimating energy expenditure expressed as metabolic equivalents (MET) and intensity classification from cut points (Sasaki et al., 2011). Notably, cut points for energy expenditure in higher-weight adults based on walking have been reported as 3454–7555 vm counts min^−1^ for moderate PA (3–6 METs) and >7555 vm counts min^−1^ for vigorous PA (>6 MET) (Howe et al., 2017).

#### Core affect

To assess core affect, the German short scale of the Multidimensional Mood Questionnaire (Steyer et al., 1997), developed and validated for momentary assessment (Wilhelm and Schoebi, 2007), was used. The scale consists of six bipolar items reflecting three basic dimensions of core affect: valence (unwell–well, discontent–content), energetic arousal (tired–awake, without energy–full of energy), and calmness (tense–relaxed, agitated–calm). The score for each subscale is the mean of the two item scores and ranges from 0 (lowest) to 100 (highest).

### Data analysis

Raw PA data was imported into the ActiLife^®^ software (v6.13.4; ActiGraph, Pensacola, FL, USA; Additional File 1). PA data was matched with the EMA questionnaire responses using electronic date and time stamps within a Microsoft Access database. Following existing EMA studies we included PA in the *15 minutes prior* to and *15 minutes following* the affect assessment. This measurement interval was also chosen for methodological reasons, as the EMA was scheduled to be at least 30 minutes apart, thus preventing the PA data from being considered more than once. Proceeding from the time stamps of the questionnaire (i.e. opening and completion), PA was calculated by aggregating the mean VM from *15 minutes prior* to (i.e. 15 minutes before the questionnaire was opened) and *15 minutes following* (starting from the time the questionnaire was completed) each completed EMA (Additional File 1). EMA/PA data, participant data (i.e. age, sex, BMI), and time variables (i.e. weekend/weekday, time of the day, day in the study, time from beginning of the study) were merged.

### Statistical analysis

R (R Core Team, 2021) and RStudio (RStudio Team, 2021) were used for all analyses (Additional File 1). Multilevel models were calculated with the repeated measurements (level 1) nested within participants (level 2). Four separate models were calculated to investigate the influence of PA in the *15 minutes prior* to the EMA on affect (H1.1–1.3) as well as the influence of affect on PA in the *15 minutes following* the assessment (H2.1–2.3).

All continuous predictors (PA in the *15 minutes prior*, valence, calmness, energetic arousal), were centered at the person-mean (Hoffman and Stawski, 2009) and included into the respective models at level 1 as fixed effects. Random effects for each predictor were included in the model. Non-significant random effects were excluded, resulting in different models for the predictors. Next, we entered a series of variables at level 1 as fixed effects to control for timely and diurnal variations (Additional File 1). PA in the *15 minutes prior* to the prompt was added as a control variable at level 1 for the PA model. The person-mean of the respective predictor and sex, BMI (kg/m^2^), and age in years were added at level 2 into the models. Variables were only included in the final models if they improved the model-fit (Additional File 1). Level for significance was set a priori to α < 0.05.

## Results

Participant’s characteristics and the mean of the predictor and outcome variables are shown in Table 1. Attrition rates are displayed in Additional File 2.

**Table 1.** Descriptive data of all participants and observations included in the analysis.

	Female (*n* = 107)		Male (*n* = 50)	
Age in years, *M* (SD)	47.0 (12.9)		51.0 (9.5)	
Body mass index, *M* (SD)	33.2 (3.7)		32.4 (3.9)	
	15 minutes prior	15 minutes following	15 minutes prior	15 minutes following
Vector magnitude, *M* (SD)	765 (772)	628 (736)	762 (873)	669 (809)
Valence, *M* (SD)	68.5 (24.5)	68.6 (24.6)	74.4 (23.3)	74.4 (23.5)
Energetic arousal, *M* (SD)	56.8 (26.9)	57.6 (26.7)	66.7 (25.5)	67.6 (25.2)
Calmness, *M* (SD)	64.9 (25.2)	64.6 (25.2)	68.7 (25.7)	68.5 (26.0)

Displayed are the means (*M*) and standard deviations (SD) for the parameters age and body mass index, as well as the mean and standard deviations of the 7-day assessment of the variables vector magnitude, valence, energetic arousal, and calmness separated by sex (female and male) and time period (15 minutes prior and 15 minutes following). A total of *N* = 157 participants and *n* = 4807 observations (15 minutes prior to the assessment) and *n* = 4276 observations (15 minutes following the assessment) were included in the analysis.

### Affect following physical activity

Within-person results indicate no significant relationship between PA and valence (Table 2). As hypothesized, PA was associated with higher energetic arousal (β = 0.08, *p* < 0.001) and lower calmness (β = −0.04, *p* = 0.035) within-persons. A 1-point increase in PA above the person-mean in the *15 minutes prior* the assessment of affect was related to an average increase of 0.0027 higher energetic arousal, and a decrease of −0.0012 calmness. Between-person results showed a significant effect between PA and energetic arousal (β = 0.10, *p* = 0.014), indicating that participants with higher PA than the average person show higher values of energetic arousal (Additional File 2).

**Table 2.** Multilevel model examining the influence of physical activity on valence, energetic arousal, and calmness.

	Valence					Energetic arousal					Calmness				
	*B*	β	95% CI	Std. 95% CI	*p*	*B*	β	95% CI	Std. 95% CI	*p*	*B*	β	95% CI	Std. 95% CI	*p*
Intercept	64.47	−0.02	57.81–71.13	−0.11 to 0.07	<**0.001**	56.73	−0.00	50.34–63.11	−0.08 to 0.07	<**0.001**	59.68	−0.01	52.40–66.95	−0.10 to 0.08	<**0.001**
VM (within)	0.0004	0.01	−0.0007 to 0.0014	−0.02 to 0.04	0.503	0.0027	0.08	0.0019–0.0035	0.05–0.10	<**0.001**	−0.0012	−0.04	−0.0024 to −0.0001	−0.07 to −0.00	**0.035**
VM (between)	0.0036	0.04	−0.0046 to 0.0117	−0.05 to 0.13	0.388	0.0097	0.10	0.0020–0.0174	0.02–0.17	**0.014**	0.0008	0.01	−0.0080 to 0.0096	−0.08 to 0.10	0.856
Weekday/weekend	3.66	0.07	2.38–4.94	0.04–0.09	<**0.001**						4.99	0.09	3.68–6.30	0.06–0.11	<**0.001**
Sex	5.60	0.11	0.82–10.38	0.02–0.20	**0.022**	9.69	0.17	5.17–14.22	0.09–0.25	<**0.001**	5.33	0.10	0.22–10.45	0.00–0.19	**0.041**
Age (in years)	0.21	0.10	0.02–0.39	0.01–0.20	**0.028**	0.24	0.11	0.07–0.42	0.03–0.19	**0.007**					
Afternoon						−5.30	−0.09	−6.88 to −3.73	−0.12 to −0.07	<**0.001**	1.66	0.03	0.20–3.12	0.00–0.06	**0.026**
Evening						−15.96	−0.28	−17.56 to −14.36	−0.31 to −0.25	<**0.001**	5.40	0.10	3.92–6.88	0.07–0.13	<**0.001**
*Random effects*															
σ^2^	396.03					488.64					411.53				
τ_00id_	179.37					155.68					212.71				
τ_11_	<0.01_id.VM_										<0.01_id.VM_				
ρ_01_	0.04_id_										0.01_id_				
ICC	0.32					0.32					0.35				
N_id_	157					157					157				
Observations	4807					4807					4807				

Displayed are the within-person results of the person-mean centered variable vector magnitude (VM) 15 minutes prior to the assessment, the control variables weekday/weekend (weekday = 0, weekend = 1), and the dummy coded control variables morning (reference), afternoon, and evening on valence, energetic arousal, and calmness (all scaled 0–100). Additionally, the between-person results of the vector magnitude, sex (0 = female, 1 = male), and the grand mean centered variable age (in years) on valence, energetic arousal, and calmness are shown. All results are displayed using the raw Beta (B), the standardized Beta (β), 95% confidence intervals (CI), and standardized (std.) 95% CI. Additionally, the within-person variance (σ^2^), the between-person variance (τ00 id), (τ11), (ρ01), the intraclass correlation coefficient (ICC), the number of participants (Nid), and the number of observations are displayed. Bold font reflects statistically significant effects.

### Physical activity following affect

Within-person results indicated no significant relationship between PA and valence (Table 3). As hypothesized, energetic arousal was associated with higher PA in the *following 15 minutes* (β = 0.09, *p* < 0.001) and lower calmness (β = −0.05, *p* = 0.007) within-persons. A 1-point increase in energetic arousal and calmness above the person-mean was associated with an average increase of 3.01 higher, and 2.03 lower PA during the *following 15 minutes*.

**Table 3.** Multilevel model analysis examining the influences of valence, energetic arousal, and calmness on physical activity.

	Vector magnitude (15 minutes following)				
	*B*	*β*	95% CI	Std. 95% CI	*p*
Intercept	481.35	−0.01	298.61–664.08	−0.06 to 0.05	<**0.001**
Valence (within)	0.64	0.02	−1.12 to 2.41	−0.03 to 0.06	0.477
Energetic arousal (within)	3.01	0.09	1.95–4.07	0.06–0.12	<**0.001**
Calmness (within)	−2.03	−0.05	−3.50 to −0.56	−0.09 to −0.02	**0.007**
VM 15 minutes prior	0.26	0.26	0.21–0.31	0.21–0.31	<**0.001**
Valence (between)	0.04	0.00	−6.39 to 6.47	−0.12 to 0.12	0.991
Energetic arousal (between)	2.66	0.05	−1.28 to 6.61	−0.02 to 0.13	0.185
Calmness (between)	−0.16	−0.00	−5.08 to 4.77	−0.10 to 0.10	0.951
BMI	−11.03	−0.06	−20.80 to −1.25	−0.11 to −0.01	**0.027**
*Random effects*					
σ^2^	450,984.42				
τ_00id_	40,603.38				
τ_11 valence (within)_	10.01				
τ_11 VM 15 minutes prior_	0.05				
ρ_01_	0.48				
	0.45				
ICC	0.14				
N_id_	157				
Observations	4276				
*R*^2^/Conditional *R*^2^	0.089/0.215				

Displayed are the within-person results of the person-mean centered variables valence, energetic arousal, and calmness, and the within-person control variable vector magnitude (VM) 15 minutes prior to the assessment on vector magnitude 15 minutes following the assessment. Additionally, the between-person results of person mean valence, energetic arousal, calmness, and grand mean centered BMI. All results are displayed using the raw Beta (B), the standardized Beta (β), 95% confidence intervals (CI), and standardized (std.) 95% CI. Additionally, the within-person variance (σ^2^), the between-person variance (τ00 id), (τ11 ID.val), (τ11 ID.vm_b15), (ρ01), the intraclass correlation coefficient (ICC), the number of participants (N id), and the number of observations are displayed. Bold font reflects statistically significant effects.

## Discussion

The primary aim of the present secondary analysis was to investigate the bi-directional association between device-based measured PA and self-reported levels of valence, energetic arousal, and calmness in everyday life in a sample of individuals with higher body weight on the within-person level. The findings support the hypothesis of a bi-directional association between PA in daily life and energetic arousal (H1.2, H2.2) and calmness (H1.3, H2.3), whereas no evidence was found for valence (H1.1, H2.1).

This null finding for valence is unexpected because from a theoretical perspective, positively valenced affect is assumed to influence the cognition, perception, and behavior of individuals insofar that beneficial resources are expanded and positive goals (i.e. health promotion) are pursued (Lyubomirsky et al., 2005; Williams and Evans, 2014). According to Fredrickson (2001), this broaden and built state is needed to prepare oneself for future challenges. In the other direction, PA is assumed to be associated with the release of mood-enhancing neurotransmitters (i.e. serotonin) and protective effects on the stress-regulation system (Basso and Suzuki, 2017). These theoretical assumptions are strengthened by prior EMA studies investigating the reciprocal relationship between PA and valence in non-clinical samples as well as in individuals with higher body weight across varying time frames (Carels et al., 2007; Emerson et al., 2018; Liao et al., 2015). An explanation for the deviating results could be the wide variety of study designs and assessments of affective valence used (Kim et al., 2020). Emerson et al. (2018) found bi-directional associations between more positive valence and higher PA at the day level. In this study, different measures for PA (i.e. self-report) and affect (i.e. feeling scale) were used which might explain the different results (Silveira et al., 2022). Another reason could be that the positive effect of PA on valence and vice versa might hold over the course of the day in higher-weight individuals but not immediately as shown in the general population (Bossmann et al., 2013; Williams et al., 2012). Our findings are comparable to those of Kanning et al. (2015), who also found that PA in the 10 minutes prior did not predict valence in older adults. Interestingly, they found that the relationship was moderated by BMI, indicating that individuals with higher BMI scores were more likely to experience higher levels of unpleasantness when they were physically active. These findings combined with our results match the considerations of Ekkekakis et al. (2016) who examined the divergent determinants of exercise in individuals with a BMI greater or equal 25 compared to “normal-weight” counterparts. In their review, they proposed a conceptual framework which describes that the greater the body weight, the more physical and psychological discomfort arises and consequently a decrease in motivation to engage in PA. In comparison to “normal-weight” counterparts, higher body-weight individuals seem to experience less pleasantness from exercise (Ekkekakis et al., 2016). Based on our results, we assume that even though the perceived consequences of acute everyday activity are unlikely as severe as in the context of structured exercise, the conscious decision to be physically active throughout the day (i.e. getting up at work, taking the stairs) could be rendered by past affective experiences in the context of exercise. Following a dual-process decision making system (Bryan et al., 2017; Hofmann et al., 2008; Williams and Evans, 2014), this process could get impeded by the affect-driven impulsive pathway which relies on interoceptive cues and, does not distinguish between different forms of movement. If this assumption can be confirmed in further experimental and controlled study designs within comparable samples, time, and PA outcomes, it may provide a possible mechanism for why people with higher body weight show lower levels of everyday activity.

As hypothesized, higher energetic arousal was reported by the participants whenever they were more physically active in the *15 minutes prior* to the assessment (H1.2). The reverse analysis showed that higher energetic arousal was associated with being more physically active in the *15 minutes following* the assessment (H2.2). Comparable results were found in previous studies investigating adults with “normal-weight” and a range of PA outcomes and time frames (Liao et al., 2015; Reichert et al., 2017) as well as for adults with higher body weight that used self-reported measures of PA within one single day (Emerson et al., 2018). These finding are reasonable because behavioral theories assume that positively valenced experiences are more likely to be repeated, thus reinforcing future PA behavior in everyday life (Stevens et al., 2020). However, the finding is particularly interesting in light of the fact that we detected no positive association between valence and PA. In our sample, higher levels of PA led to a high-activation (i.e. feeling awake and full of energy) but not to more pleasantness. To clarify these findings, it would be necessary to assess if other variables, such as the context or the degree of self-determination, are responsible for this association (Kanning et al., 2021). To establish a causal interpretation and inform future PA interventions, a promising approach would be to experimentally modify participants’ energetic arousal and analyze the effect on PA. If this association proves to be causal then feeling tired and lacking energy may be a barrier for PA uptake.

We found evidence for a significant negative bi-directional association between PA and calmness. If individuals with higher body-weight were more physically active, they felt more agitated (i.e. less calm and relaxed; H1.3), and if participants felt calmer they were less physically active (H2.3). This finding is consistent with previous EMA studies investigating the influence of calmness in populations with normal weight (Kanning et al., 2015; Reichert et al., 2016, 2017). Reichert et al. (2016) showed that this negative within-person influence is still evident up to 140 minutes. The extent to which PA and calmness are interdependent or if further variables moderate this association is still unclear. It could be, for example, that higher levels of PA are associated with higher levels of stress (Stults-Kolehmainen and Sinha, 2014) (e.g. running to catch a train ride) or internalized weight stigma (Carels et al., 2019) (e.g. walking up stairs while being watched by others). When developing PA interventions, researchers and practitioners should inform participants about the acute effects of PA (i.e. restlessness) and the long-term beneficial effects on well-being. Consideration of the optimal timing of PA is important to improve well-being (i.e. not at the end of the day when levels of calmness tend to be lower) (Kanning et al., 2015).

The strengths of our investigation are that it includes a large sample of the understudied population of higher-weight individuals, combines device-based measured PA with EMA, and follows a pre-registered design (Liao et al., 2016). Nonetheless, some limitations must be considered when interpreting the results of this paper. First, PA data were not distinguished between exercise and non-exercise PA even though previous studies showed distinct associations to core affect (Reichert et al., 2017). Given the limited studies on participants with higher body weight, this study included only the raw measure of activity counts. Future studies should delve deeper and include other PA measures (i.e. light, moderate, and vigorous PA, step count) and contextual variables. Second, participants had unlimited time to respond to the prompts because the program had no timeout feature. Time was accounted for in the data processing, but it led to missing data. Including timeout for prompts could reduce participant burden in future research. Third, time windows of 15 minutes were chosen for the analysis of the bi-directional association of PA and affect according to previous literature (Bourke et al., 2021; Liao et al., 2017; Schwerdtfeger et al., 2008). Given the insufficient knowledge about the temporal course of these associations, various time windows should be explored in future studies (Kim et al., 2020; Reichert et al., 2016). Fourth, some data collection took place during the COVID-19 pandemic. Because of variations in the timing of the study enrollment and data assessment, we did not systematically examine the potential influence of different restrictions in different regions. Finally, it must be taken into account that no independent power analysis was performed for the present secondary analysis. Future studies should perform simulation-based multilevel power analysis to ensure adequate sample size.

## Conclusions

Overall, the results suggest distinct acute bi-directional within-person associations between the dimensions of core affect and PA among adults with higher body weight in everyday life. Although more research is needed to confirm these findings, we suggest two important implications as a starting point for researchers and practitioners that might inform the development and design of (digital) interventions targeting PA engagement.

First, the motivation to engage in long-term everyday PA among higher-weight individuals could be enhanced by delivering health communication messages about the benefits of PA that go beyond the weight-related health outcomes. Psychoeducational material should be provided that highlights the affect-regulation potential of everyday PA. Moreover, our finding that being physically active led to no enhancement in feeling better contradicts the narrative often portrayed in PA programs (“move more and you will feel better”). Hence, this association might not apply as an acute effect to higher-weight individuals and should thus be presented with caution. Such messages could establish a discrepancy between the expectations of PA and the actual experience of PA. Instead, participants’ attention should be drawn to feelings of energy and agitation (i.e. psychoeducation and self monitoring after PA engagement), which occur even after short periods of PA and which might increase the adaption of a habitual PA lifestyle.

Second, our results suggest that feeling more energized and agitated predicted acute PA engagement in a sample of higher-weight individuals. These findings could imply that interventions designed to foster PA might be more effective when they deliver brief affect-enhancing strategies that target the modification of energetic arousal and calmness in real-time. Higher-weight individuals might benefit from guided mental imagery tasks about PA engagement or the confrontation with auditory or visual stimuli (i.e. high arousal rhythmic music, positive media content). Additionally, situations in which an individual feels more energetic and less calm might represent an opportune moment for PA interventions (i.e. recommend PA when ratings of energetic arousal are high and ratings of calmness are low). The timing of such personalized acute interventions could be identified by affect focused assessments and the identification of individual patterns of affect fluctuation (i.e. just-in-time adaptive interventions).

Replication of the present evidence on distinct influences of core affect and PA within other (more diverse) samples (i.e. BMI, gender, race, no treatment enrollment) is needed to generalize these findings and confirm causality. Likewise, we encourage further studies to implement experimental approaches (e.g. manipulation of energetic arousal) and to investigate factors that might moderate the nature of the reciprocal associations in individuals with higher body weight (e.g. internalized weight bias, physical limitations, context, intensity of PA, self-determination, self-efficacy, depression).

## Supplemental Material

sj-docx-1-hpq-10.1177_13591053241228202 – Supplemental material for Bi-directional associations of core affect and physical activity in adults with higher body weight: An ecological momentary assessment study Supplemental material, sj-docx-1-hpq-10.1177_13591053241228202 for Bi-directional associations of core affect and physical activity in adults with higher body weight: An ecological momentary assessment study by Caroline Seiferth, Janis Fiedler, Tanja Färber, Magdalena Pape, Stefanie Schroeder, Stephan Herpertz, Sabine Steins-Loeber and Jörg Wolstein in Journal of Health Psychology

sj-docx-2-hpq-10.1177_13591053241228202 – Supplemental material for Bi-directional associations of core affect and physical activity in adults with higher body weight: An ecological momentary assessment study Supplemental material, sj-docx-2-hpq-10.1177_13591053241228202 for Bi-directional associations of core affect and physical activity in adults with higher body weight: An ecological momentary assessment study by Caroline Seiferth, Janis Fiedler, Tanja Färber, Magdalena Pape, Stefanie Schroeder, Stephan Herpertz, Sabine Steins-Loeber and Jörg Wolstein in Journal of Health Psychology

sj-docx-3-hpq-10.1177_13591053241228202 – Supplemental material for Bi-directional associations of core affect and physical activity in adults with higher body weight: An ecological momentary assessment study Supplemental material, sj-docx-3-hpq-10.1177_13591053241228202 for Bi-directional associations of core affect and physical activity in adults with higher body weight: An ecological momentary assessment study by Caroline Seiferth, Janis Fiedler, Tanja Färber, Magdalena Pape, Stefanie Schroeder, Stephan Herpertz, Sabine Steins-Loeber and Jörg Wolstein in Journal of Health Psychology

## References

[bibr1-13591053241228202] Aadland E Ylvisåker E (2015) Reliability of the actigraph GT3X+ accelerometer in adults under free-living conditions. PLoS One 10(8): e0134606. 10.1371/journal.pone.0134606 PMC4537282 26274586

[bibr2-13591053241228202] Baillot A Chenail S Barros Polita N , et al (2021) Physical activity motives, barriers, and preferences in people with obesity: A systematic review. PLoS One 16(6): e0253114. 10.1371/journal.pone.0253114 PMC8221526 34161372

[bibr3-13591053241228202] Basso JC Suzuki WA (2017) The effects of acute exercise on mood, cognition, neurophysiology, and neurochemical pathways: A review. Brain Plasticity 2(2): 127–152. 29765853 10.3233/BPL-160040 PMC5928534

[bibr4-13591053241228202] Berger BG Darby LA Owen DR , et al (2023) “Feeling Good” after exercise during a weight loss program: Subjective well-being in support of a hedonic paradigm. Perceptual and Motor Skills 130: 434–460. 36176046 10.1177/00315125221130444

[bibr5-13591053241228202] Bossmann T Kanning M Koudela-Hamila S , et al (2013) The association between short periods of everyday life activities and affective states: A replication study using ambulatory assessment. Frontiers in Psychology 4: 102. 23596426 10.3389/fpsyg.2013.00102 PMC3625722

[bibr6-13591053241228202] Bourke M Hilland TA Craike M (2021) A systematic review of the within-person association between physical activity and affect in children’s and adolescents’ daily lives. Psychology of Sport and Exercise 52: 101825.

[bibr7-13591053241228202] Brose A Schmiedek F Gerstorf D , et al (2020) The measurement of within-person affect variation. Emotion 20(4): 677–699. 31008618 10.1037/emo0000583

[bibr8-13591053241228202] Bryan AD Jakicic JM Hunter CM , et al (2017) Behavioral and psychological phenotyping of physical activity and sedentary behavior: Implications for weight management. Obesity 25(10): 1653–1659. 28948719 10.1002/oby.21924 PMC5657446

[bibr9-13591053241228202] Burchartz A Anedda B Auerswald T , et al (2020) Assessing physical behavior through accelerometry – State of the science, best practices and future directions. Psychology of Sport and Exercise 49: 101703.

[bibr10-13591053241228202] Carels RA Coit C Young K , et al (2007) Exercise makes you feel good, but does feeling good make you exercise? An examination of obese dieters. Journal of Sport and Exercise Psychology 29(6): 706–722. 18089900 10.1123/jsep.29.6.706

[bibr11-13591053241228202] Carels RA Rossi J Solar C , et al (2019) An ecological momentary assessment of weight stigma among weight loss participants. Journal of Health Psychology 24(9): 1155–1166. 28810406 10.1177/1359105317692855

[bibr12-13591053241228202] Carraça EV Encantado J Battista F , et al (2021) Effect of exercise training on psychological outcomes in adults with overweight or obesity: A systematic review and meta-analysis. Obesity Reviews 22: e13261. 10.1111/obr.13261 PMC8365728 33960106

[bibr13-13591053241228202] Cassidy S Chau JY Catt M , et al (2017) Low physical activity, high television viewing and poor sleep duration cluster in overweight and obese adults; a cross-sectional study of 398,984 participants from the UK Biobank. International Journal of Behavioral Nutrition and Physical Activity 14(1): 57. 28454540 10.1186/s12966-017-0514-y PMC5408822

[bibr14-13591053241228202] Cleland I Kikhia B Nugent C , et al (2013) Optimal placement of accelerometers for the detection of everyday activities. Sensors (Basel, Switzerland) 13(7): 9183–9200. 23867744 10.3390/s130709183 PMC3758644

[bibr15-13591053241228202] Conroy DE Berry TR (2017) Automatic affective evaluations of physical activity. Exercise and Sport Sciences Reviews 45(4): 230–237. 28704217 10.1249/JES.0000000000000120

[bibr16-13591053241228202] Ekkekakis P (2013) The Measurement of Affect, Mood, and Emotion: A Guide for Health-Behavioral Research. Cambridge and New York, NY: Cambridge University Press.

[bibr17-13591053241228202] Ekkekakis P Brand R (2019) Affective responses to and automatic affective valuations of physical activity: Fifty years of progress on the seminal question in exercise psychology. Psychology of Sport and Exercise 42: 130–137.

[bibr18-13591053241228202] Ekkekakis P Lind E (2006) Exercise does not feel the same when you are overweight: The impact of self-selected and imposed intensity on affect and exertion. International Journal of Obesity 30: 652–660. 16130028 10.1038/sj.ijo.0803052

[bibr19-13591053241228202] Ekkekakis P Lind E Vazou S (2010) Affective responses to increasing levels of exercise intensity in normal-weight, overweight, and obese middle-aged women. Obesity 18(1): 79–85. 19556979 10.1038/oby.2009.204

[bibr20-13591053241228202] Ekkekakis P Vazou S Bixby WR , et al (2016) The mysterious case of the public health guideline that is (almost) entirely ignored: Call for a research agenda on the causes of the extreme avoidance of physical activity in obesity. Obesity Reviews 17(4): 313–329. 26806460 10.1111/obr.12369

[bibr21-13591053241228202] Emerson JA Dunsiger S Williams DM (2018) Reciprocal within-day associations between incidental affect and exercise: An EMA study. Psychology and Health 33(1): 130–143. 28665227 10.1080/08870446.2017.1341515 PMC5738286

[bibr22-13591053241228202] Emerson JA Williams DM (2015) The multifaceted relationship between physical activity and affect. Social and Personality Psychology Compass 9(8): 419–433.

[bibr23-13591053241228202] Engel SG Crosby RD Thomas G , et al (2016) Ecological momentary assessment in eating disorder and obesity research: A review of the recent literature. Current Psychiatry Reports 18(4): 37. 26893235 10.1007/s11920-016-0672-7

[bibr24-13591053241228202] Forster AK Richards EA Foli KJ , et al (2021) Influence of affect on physical activity: An integrative review. Clinical Nursing Research 30(7): 934–949. 33111569 10.1177/1054773820968039

[bibr25-13591053241228202] Fredrickson BL (2001) The role of positive emotions in positive psychology. The broaden-and-build theory of positive emotions. American Psychologist 56(3): 218–226. 11315248 10.1037//0003-066x.56.3.218 PMC3122271

[bibr26-13591053241228202] Hamer O Larkin D Relph N , et al (2021) Fear-related barriers to physical activity among adults with overweight and obesity: A narrative synthesis scoping review. Obesity Reviews 22(11): e13307. 10.1111/obr.13307 34170596

[bibr27-13591053241228202] Hoffman L Stawski RS (2009) Persons as contexts: Evaluating between-person and within-person effects in longitudinal analysis. Research in Human Development 6(2–3): 97–120.

[bibr28-13591053241228202] Hofmann W Friese M Wiers RW (2008) Impulsive versus reflective influences on health behavior: A theoretical framework and empirical review. Health Psychology Review 2(2): 111–137.

[bibr29-13591053241228202] Howe CCF Moir HJ Easton C (2017) Classification of physical activity cut-points and the estimation of energy expenditure during walking using the GT3X+ accelerometer in overweight and obese adults. Measurement in Physical Education and Exercise Science 21(3): 127–133.

[bibr30-13591053241228202] Hulens M Vansant G Claessens AL , et al (2003) Predictors of 6-minute walk test results in lean, obese and morbidly obese women. Scandinavian Journal of Medicine and Science in Sports 13(2): 98–105. 12641641 10.1034/j.1600-0838.2003.10273.x

[bibr31-13591053241228202] Hyde AL Conroy DE Pincus AL , et al (2011) Unpacking the feel-good effect of free-time physical activity: Between- and within-person associations with pleasant–activated feeling states. Journal of Sport and Exercise Psychology 33(6): 884–902. 22262710 10.1123/jsep.33.6.884 PMC4144026

[bibr32-13591053241228202] Kanning M Ebner-Priemer U Schlicht W (2013) How to investigate within-subject associations between physical activity and momentary affective states in everyday life: A position statement based on a literature overview. Frontiers in Psychology 4: 187. 23641221 10.3389/fpsyg.2013.00187 PMC3638123

[bibr33-13591053241228202] Kanning M Ebner-Priemer U Schlicht W (2015) Using activity triggered e-diaries to reveal the associations between physical activity and affective states in older adult’s daily living. International Journal of Behavioral Nutrition and Physical Activity 12: 111. 26377553 10.1186/s12966-015-0272-7 PMC4573919

[bibr34-13591053241228202] Kanning M Niermann C Ebner-Primer U , et al (2021) The context matters - not all prolonged sitting bouts are equally related to momentary affective states: An ambulatory assessment with sedentary-triggered E-diaries. International Journal of Behavioral Nutrition and Physical Activity 18(1): 106. 34391442 10.1186/s12966-021-01170-3 PMC8364093

[bibr35-13591053241228202] Kanning MK Schoebi D (2016) Momentary affective states are associated with momentary volume, prospective trends, and fluctuation of daily physical activity. Frontiers in Psychology 7: 744. 27242643 10.3389/fpsyg.2016.00744 PMC4876120

[bibr36-13591053241228202] Kerrigan SG Schumacher L Manasse SM , et al (2020) The association between negative affect and physical activity among adults in a behavioral weight loss treatment. Psychology of Sport and Exercise 47: 101507. 32440257 10.1016/j.psychsport.2019.03.010 PMC7241248

[bibr37-13591053241228202] Kim J Conroy DE Smyth JM (2020) Bidirectional associations of momentary affect with physical activity and sedentary behaviors in working adults. Annals of Behavioral Medicine 54(4): 268–279. 31613961 10.1093/abm/kaz045

[bibr38-13591053241228202] Koch ED Tost H Braun U , et al (2018) Mood dimensions show distinct within-subject associations with non-exercise activity in adolescents: An ambulatory assessment study. Frontiers in Psychology 9: 268. 29563889 10.3389/fpsyg.2018.00268 PMC5850094

[bibr39-13591053241228202] Ladwig MA Sciamanna CN Auer BJ , et al (2021) When American adults do move, how do they do so? Trends in physical activity intensity, type, and modality: 1988-2017. Journal of Physical Activity and Health 18(10): 1181–1198. 34470912 10.1123/jpah.2020-0424

[bibr40-13591053241228202] Le F Yap Y Tung NYC , et al (2022) The associations between daily activities and affect: A compositional isotemporal substitution analysis. International Journal of Behavioral Medicine 29(4): 456–468. 34608593 10.1007/s12529-021-10031-z

[bibr41-13591053241228202] Liao Y Chou C-P Huh J , et al (2017) Examining acute bi-directional relationships between affect, physical feeling states, and physical activity in free-living situations using electronic ecological momentary assessment. Journal of Behavioral Medicine 40(3): 445–457. 27766481 10.1007/s10865-016-9808-9 PMC5398956

[bibr42-13591053241228202] Liao Y Shonkoff ET Dunton GF (2015) The acute relationships between affect, physical feeling states, and physical activity in daily life: A review of current evidence. Frontiers in Psychology 6: 1975. 26779049 10.3389/fpsyg.2015.01975 PMC4688389

[bibr43-13591053241228202] Liao Y Skelton K Dunton G , et al (2016) A systematic review of methods and procedures used in ecological momentary assessments of diet and physical activity research in youth: An adapted STROBE Checklist for Reporting EMA Studies (CREMAS). Journal of Medical Internet Research 18(6): e151. 10.2196/jmir.4954 PMC4933800 27328833

[bibr44-13591053241228202] Lyubomirsky S King L Diener E (2005) The benefits of frequent positive affect: Does happiness lead to success? Psychological Bulletin 131(6): 803–855. 16351326 10.1037/0033-2909.131.6.803

[bibr45-13591053241228202] Migueles JH Aadland E Andersen LB , et al (2022) GRANADA consensus on analytical approaches to assess associations with accelerometer-determined physical behaviours (physical activity, sedentary behaviour and sleep) in epidemiological studies. British Journal of Sports Medicine 56(7): 376–384. 33846158 10.1136/bjsports-2020-103604 PMC8938657

[bibr46-13591053241228202] Oppert J-M Ciangura C Bellicha A (2023) Physical activity and exercise for weight loss and maintenance in people living with obesity. Reviews in Endocrine and Metabolic Disorders 24(5): 937–949. 37142892 10.1007/s11154-023-09805-5

[bibr47-13591053241228202] Pape M Färber T Seiferth C , et al (2022) A tailored gender-sensitive mHealth weight loss intervention (I-GENDO): Development and process evaluation. JMIR Formative Research 6(10): e38480. 10.2196/38480 PMC9650578 36301614

[bibr48-13591053241228202] R Core Team (2021) R: A Language and Environment for Statistical. Vienna, Austria: R Foundation for Statistical Computing.

[bibr49-13591053241228202] Reed J Ones DS (2006) The effect of acute aerobic exercise on positive activated affect: A meta-analysis. Psychology of Sport and Exercise 7(5): 477–514.

[bibr50-13591053241228202] Reichert M Tost H Reinhard I , et al (2016) Within-subject associations between mood dimensions and non-exercise activity: An ambulatory assessment approach using repeated real-time and objective data. Frontiers in Psychology 7: 918. 27445891 10.3389/fpsyg.2016.00918 PMC4919351

[bibr51-13591053241228202] Reichert M Tost H Reinhard I , et al (2017) Exercise versus nonexercise activity: E-diaries unravel distinct effects on mood. Medicine and Science in Sports and Exercise 49(4): 763–773. 27824691 10.1249/MSS.0000000000001149

[bibr52-13591053241228202] Rhodes RE Kates A (2015) Can the affective response to exercise predict future motives and physical activity behavior? A systematic review of published evidence. Annals of Behavioral Medicine 49(5): 715–731. 25921307 10.1007/s12160-015-9704-5

[bibr53-13591053241228202] RStudio Team (2021) RStudio: Integrated Development for R. Boston, MA: RStudio, PBC.

[bibr54-13591053241228202] Ruissen GR Beauchamp MR Puterman E , et al (2022) Continuous-time modeling of the bidirectional relationship between Incidental affect and physical activity. Annals of Behavioral Medicine 56(12): 1284–1299. 35802004 10.1093/abm/kaac024 PMC9672348

[bibr55-13591053241228202] Russell JA (1980) A circumplex model of affect. Journal of Personality and Social Psychology 39(6): 1161–1178.

[bibr56-13591053241228202] Russell JA (2003) Core affect and the psychological construction of emotion. Psychological Review 110(1): 145–172. 12529060 10.1037/0033-295x.110.1.145

[bibr57-13591053241228202] Sasaki JE John D Freedson PS (2011) Validation and comparison of ActiGraph activity monitors. Journal of Science and Medicine in Sport 14(5): 411–416. 21616714 10.1016/j.jsams.2011.04.003

[bibr58-13591053241228202] Schimmack U Grob A (2000) Dimensional models of core affect: A quantitative comparison by means of structural equation modeling. European Journal of Personality 14(4): 325–345.

[bibr59-13591053241228202] Schimmack U Rainer R (2002) Experiencing activation: Energetic arousal and tense arousal are not mixtures of valence and activation. Emotion 2(4): 412–417. 12899373 10.1037/1528-3542.2.4.412

[bibr60-13591053241228202] Schwerdtfeger A Eberhardt R Chmitorz A (2008) Gibt es einen Zusammenhang zwischen Bewegungsaktivität und psychischem Befinden im Alltag? Zeitschrift für Gesundheitspsychologie 16(1): 2–11.

[bibr61-13591053241228202] Schwerdtfeger A Eberhardt R Chmitorz A , et al (2010) Momentary affect predicts bodily movement in daily life: An ambulatory monitoring study. Journal of Sport and Exercise Psychology 32: 674–693. 20980710 10.1123/jsep.32.5.674

[bibr62-13591053241228202] Seiferth C Färber T Pape M , et al (2023) Differential effects of the individualized gender-sensitive mHealth intervention I-GENDO on eating styles in individuals with overweight and obesity – A randomized controlled trial. BMC Digital Health 1(1): 46.

[bibr63-13591053241228202] Silveira EA Mendonça CR Delpino FM , et al (2022) Sedentary behavior, physical inactivity, abdominal obesity and obesity in adults and older adults: A systematic review and meta-analysis. Clinical Nutrition ESPEN 50: 63–73. 35871953 10.1016/j.clnesp.2022.06.001

[bibr64-13591053241228202] Stevens CJ Baldwin AS Bryan AD , et al (2020) Affective determinants of physical activity: A conceptual framework and narrative review. Frontiers in Psychology 11: 3366. 10.3389/fpsyg.2020.568331 PMC7735992 33335497

[bibr65-13591053241228202] Steyer R Schwenkmezger P Notz P , et al (1997) Der Mehrdimensionale Befindlichkeitsfragebogen MDBF [Multidimensional Mood Questionnaire]. Göttingen: Hogrefe.

[bibr66-13591053241228202] Stults-Kolehmainen MA Sinha R (2014) The effects of stress on physical activity and exercise. Sports Medicine 44(1): 81–121. 24030837 10.1007/s40279-013-0090-5 PMC3894304

[bibr67-13591053241228202] Thayer RE (1990) The Biopsychology of Mood and Emotion. New York, NY: Oxford University Press.

[bibr68-13591053241228202] Thedinga HK Zehl R Thiel A (2021) Weight stigma experiences and self-exclusion from sport and exercise settings among people with obesity. BMC Public Health 21(1): 565. 33752645 10.1186/s12889-021-10565-7 PMC7983352

[bibr69-13591053241228202] Unick JL Michael JC Jakicic JM (2012) Affective responses to exercise in overweight women: Initial insight and possible influence on energy intake. Psychology of Sport and Exercise 13(5): 528–532. 24039545 10.1016/j.psychsport.2012.02.012 PMC3772527

[bibr70-13591053241228202] Unick JL Strohacker K Papandonatos GD , et al (2015) Examination of the consistency in affective response to acute exercise in overweight and obese women. Journal of Sport and Exercise Psychology 37(5): 534–546. 26524099 10.1123/jsep.2015-0104 PMC4724861

[bibr71-13591053241228202] Watson D Tellegen A (1985) Toward a consensual structure of mood. Psychological Bulletin 98(2): 219–235. 3901060 10.1037//0033-2909.98.2.219

[bibr72-13591053241228202] Wilhelm P Schoebi D (2007) Assessing mood in daily life. European Journal of Psychological Assessment 23(4): 258–267.

[bibr73-13591053241228202] Williams DM Dunsiger S Jennings EG , et al (2012) Does affective valence during and immediately following a 10-Min walk predict concurrent and future physical activity? Annals of Behavioral Medicine 44(1): 43–51. 22532005 10.1007/s12160-012-9362-9 PMC5718347

[bibr74-13591053241228202] Williams DM Evans DR (2014) Current emotion research in health behavior science. Emotion Review 6(3): 277–287. 10.1177/1754073914523052 PMC12975116 41815188

[bibr75-13591053241228202] Williams DM Rhodes RE Conner MT (2019) Conceptualizing and intervening on affective determinants of health behaviour. Psychology and Health 34(11): 1267–1281. 31617431 10.1080/08870446.2019.1675659

