# Supplementary material for: Bi-directional associations of core affect and physical activity in adults with higher body weight: An ecological momentary assessment study
Source: J Health Psychol. 2024 Jan 29;29(10):1115–28. doi: 10.1177/13591053241228202 (PMC11344957; doi:10.1177/13591053241228202)
Supplement: sj-docx-1-hpq-10.1177_13591053241228202 – Supplemental material for Bi-directional associations of core affect and physical activity in adults with higher body weight: An ecological momentary assessment study [file sj-docx-1-hpq-10.1177_13591053241228202.docx]

**ADDITIONAL FILE 1**

**Title**: Bi-directional associations of core affect and physical activity in adults with overweight and obesity – An ecological momentary assessment study

**A) Additional files for the METHODS section**

- **Table S1.** Eligibility criteria of the study.

| **Inclusion Criteria** | **Exclusion Criteria** |
| --- | --- |
| - ≥18 years of age - Obesity class I or II (30.00 - 39.99 kg/m^2^) with subjectively experienced weight-related impairment OR - Overweight (25.00 - 29.99 kg/m^2^) with weight-related health problems and/or visceral adipose tissue and/or high psychosocial weight-related distress - Current motivation to lose weight - Access to a smartphone | - Current (or within the last 12 months) involvement in a structured weight loss intervention or psychotherapeutic treatment of weight-related health problems - Previous or intended bariatric surgery - Current pregnancy - Current intake of drugs that influence weight - Current substance abuse, major depression, suicidal ideation - Severe cognitive impairments - Insufficient knowledge of the German language - Binge eating disorder or bulimia nervosa - Insulin-dependent type 1 diabetes - Cancerous disease within the last 5 years |

- **Deviations from the pre-registration**

In contrast to the pre-registration, we explored no further time-dependent associations by specifying additional models with shorter time intervals of physical activity (e.g., 5 and 10 min) for a more concise focus.

- **Explanatory notes regarding the device measured physical activity and data preprocessing**

The ActiGraph® wGT3X-BT accelerometer were small-scale (4.6cm x 3.3cm x 1.5 cm), light weight (19 grams), and attached to an elastic waist belt.

Raw data was sampled at an input frequency of 30 Hz and initially stored on the device. Raw accelerations were filtered using the ‘Normal Filter’ mode and accumulated into 60-second epochs. Data was screened for non-wear periods using the waist-worn inclinometer algorithm^[[1]](#footnote-1)^ from ActiGraph® which classifies participant’s posture into standing, sitting, lying, and non-wear/off. We excluded 60-second epochs with more than 30 ‘off’ seconds (non-valid epoch).

- **Figure S1.** Flow Chart of the data preparation process.

Participants with less than 10 hours of accelerometer wear-time for three or more days (non-valid days) were excluded. We further removed participants that completed less than 17 (30%) of the 56 EMA questionnaires (non-compliant) (Reichert et al., 2016).


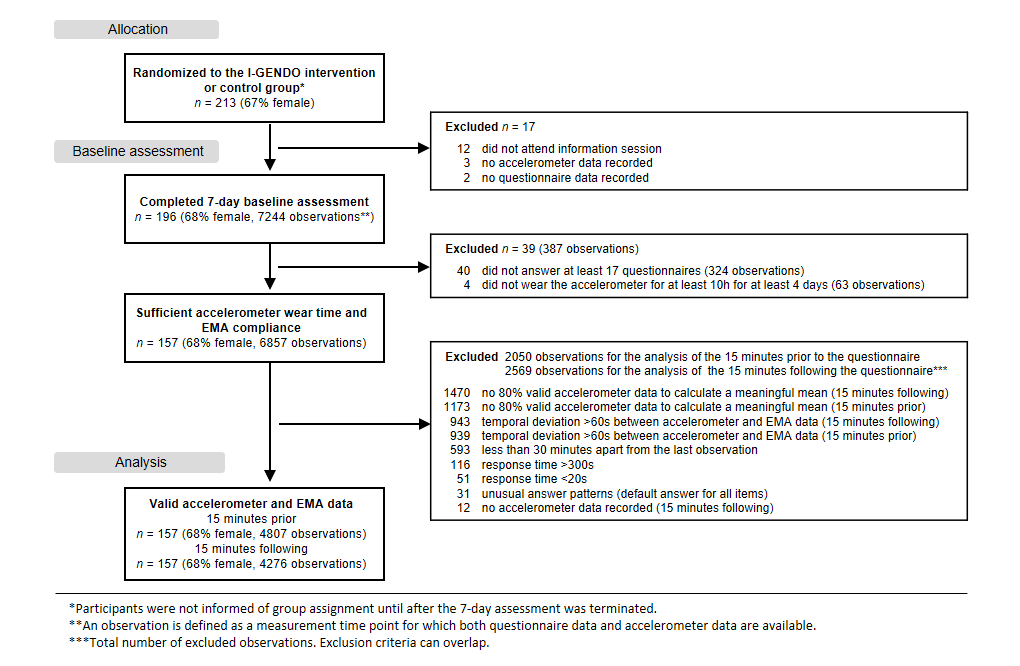


- **Figure S2.** Illustration of the predicted association between physical activity and core affect


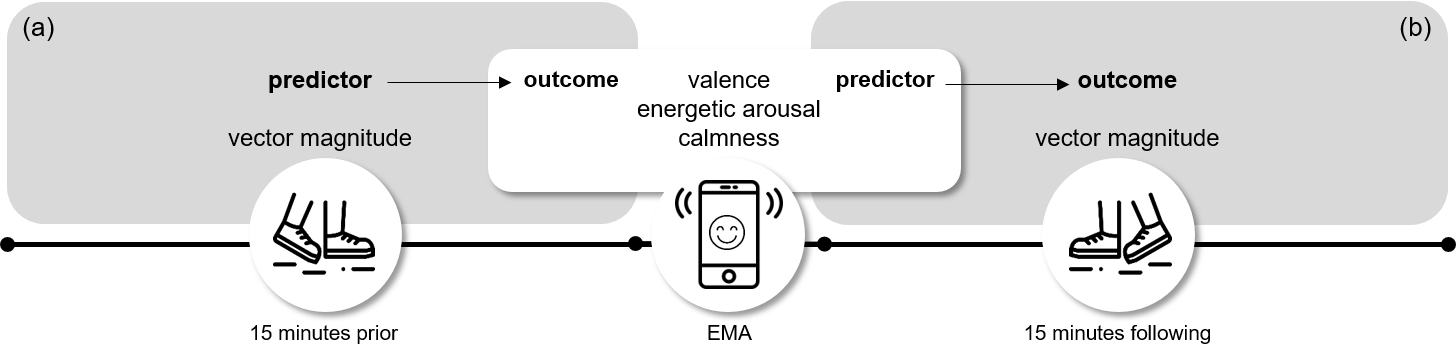


***Legend.*** *Association between physical activity (PA) and affect (a) 15 minutes prior to and (b) 15 minutes following the ecological momentary assessment (EMA). PA (i.e., vector magnitude) was assessed continuously with an accelerometer. Core affect (i.e., valence, energetic arousal, calmness) was assessed via a questionnaire on participants smartphone at random occasions eight times a day. The time needed to complete the questionnaire was not considered in the analysis.*

- **Table S2.** R packages used for all analysis.

| **Package** | **Application** | **Reference** |
| --- | --- | --- |
| ggplot2 (v 3.3.5) | to generate visualizations | Wickham, H., Chang, W., & Wickham, M. H. (2016). Package ‘ggplot2’. *Create elegant data visualisations using the grammar of graphics*. Version, 2(1), 1-189. |
| nlme (v. 3.1 – 155) | to fit multilevel models | Pinheiro, J., Bates, D., DebRoy, S., Sarkar, D., Heisterkamp, S., Van Willigen, B., & Maintainer, R. (2017). Package ‘nlme’. *Linear and nonlinear mixed effects models,* version, 3(1), 274. |
| Performance (c. 0.8.0) | to check model assumptions | Lüdecke, D., Ben-Shachar, M. S., Patil, I., Waggoner, P., & Makowski, D. (2021). performance: An R package for assessment, comparison and testing of statistical models. *Journal of Open Source Software*, 6(60). |
| robustlmm | to fit robust models | Koller, M. (2016). robustlmm: an R package for robust estimation of linear mixed-effects models*. Journal of statistical software*, 75, 1-24. |
| sjPlot (v 2.8.10) | to generate tables of regression analysis | Lüdecke, D., & Lüdecke, M. D. (2015). Package ‘sjPlot’. *R package version*, 1(9). |

- **Statistical analysis.** Checking the assumptions

Assumptions were checked by visual inspection and if they appeared to be violated, a robust model was fitted and compared to the non-robust version. No noticeable difference emerged between both versions of the models. Model fit was not improved when controlling for autocorrelation.

**Statistical analysis.** Model fit A pre-registered hierarchical approach was used for the inclusion of the control variables and the model-fit was assessed with the Akaike information criterion. Maximum likelihood estimations were used to estimate model parameters and guide the inclusion of control variables. Models including all variables of interest based on theoretical considerations were run as a sensitivity analysis. These results did not differ from the hierarchical approach and were thus not reported. For the results see <https://osf.io/p6xuf/?view_only=2e3db5614dcf4d1baaed797b359839c5>

- **Statistical analysis.** Multilevel models

All continuous predictors (PA in the 15 minutes prior, valence, calmness, energetic arousal), were centred at the person-mean (Hoffman and Stawski, 2009) and included into the respective models at level 1 as fixed effects. Random effects for each predictor were included in the model. Non-significant random effects were excluded, resulting in different models for the predictors. Next, we entered a series of variables at level 1 as fixed effects to control for timely and diurnal variations: weekday or weekend (weekday=0, weekend=1), time-of-day (morning=00:00:00–11:59:59 (reference), afternoon=12:00:00–16:59:59, evening=17:00:00–23:59:59) and day in the study (0–6). PA in the 15 minutes prior to the prompt was added as an additional control variable at level 1 for the PA model. The person-mean of the respective predictor and sex (female=0, male=1), BMI (kg/m2) and age in years (both centred at grand-mean) were added at level 2 into the models (Additional File 1). Variables were only included in the final models if they improved the model-fit. Level for significance was set a priori to α<0.05.

- **Figure S3.** Equations of the final models for the dependent variables (a) valence, calmness, energetic arousal and (b) VM in the 15 minutes following the prompt


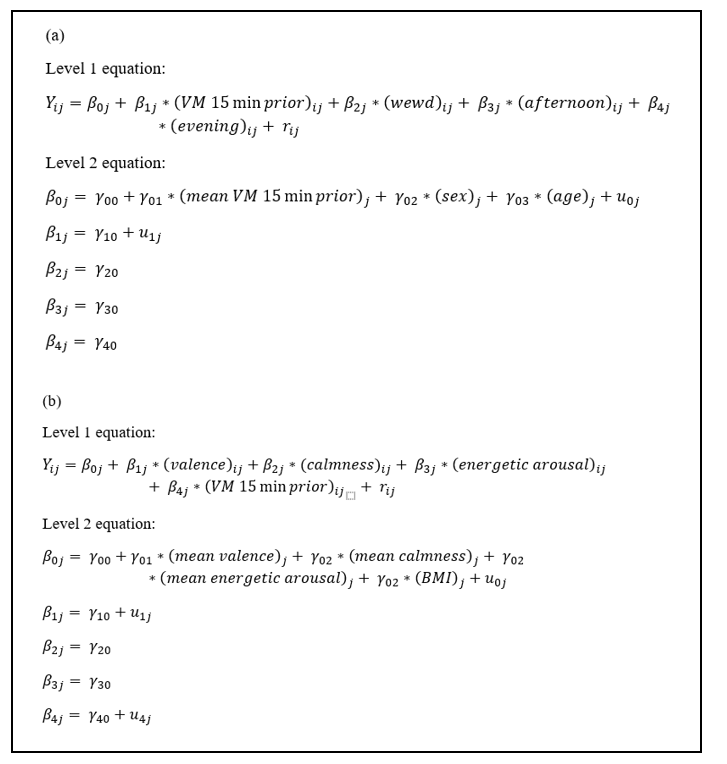


1. [https://docs.google.com/document/d/1EBAEcAL34k0ONZOgfXZPsMJJC-Uy9d49FFyPnmPH3Qc/edit?hl=en&authkey=CM--zvgE&pli=1#](https://docs.google.com/document/d/1EBAEcAL34k0ONZOgfXZPsMJJC-Uy9d49FFyPnmPH3Qc/edit?hl=en&authkey=CM--zvgE&pli=1) [↑](#footnote-ref-1)
