# Supplementary material for: Bi-directional associations of core affect and physical activity in adults with higher body weight: An ecological momentary assessment study
Source: J Health Psychol. 2024 Jan 29;29(10):1115–28. doi: 10.1177/13591053241228202 (PMC11344957; doi:10.1177/13591053241228202)
Supplement: sj-docx-2-hpq-10.1177_13591053241228202 – Supplemental material for Bi-directional associations of core affect and physical activity in adults with higher body weight: An ecological momentary assessment study [file sj-docx-2-hpq-10.1177_13591053241228202.docx]

**ADDITIONAL FILE 2**

**Title**: Bi-directional associations of core affect and physical activity in adults with overweight and obesity – An EMA study

**B) Additional files for the RESULTS section**

- **Data preprocessing**

Ninty-three of the initial 196 participants were excluded from the analysis because they did not wear the sensor for at least 10h/day for at least four days (*n* = 4; 63 observations) or answered less than 17 EMA prompts (*n* = 35; 324 observations). The remaining 157 participants answered 6857 (*M* = 43.67, *SD* = 15.88, range: 17 – 98) EMA prompts while 8792 were planned to be delivered. Further, 2050 (15 min prior) and 2569 (15 min following) observations were excluded due to practical and statistical reasons, yielding a final observation count of 4807 (15 min prior) and 4276 (15 min following).

- **Attrition rate over the course of the 7-day assessment**

On the final day of the assessments (day 7) participants completed 15% fewer EMA prompts compared to the first day of the assessment (day 1: 1056, day 7: 896).

- **Between-person results**

Intraclass correlation coefficients (ICCs) of the null models indicated that 31% (valence), 33% (calmness), 25% (energetic arousal) and 7% (VM) were due to between-person differences.

AFFECT FOLLOWING PHYSICAL ACTIVITY

The day of the week (week day, weekend) showed a significant effect on the ratings of affect. This results means that on a Saturday or Sunday participants rating of valence and calmness increased by 3.66 and 4.99 respectively. In addition, the rating of all three subscales of affect was significantly higher for male than for female participants (valence: β=0.11, *p*=0.02, energetic arousal: β=0.17, *p*<0.001, calmness: β=0.10, *p*=0.04) and significant age differences were found for valence and energetic arousal. Being one year older than the group average was associated with higher values (valence: β=0.10, *p*=0.028, energetic arousal: β=0.11, *p*=0.007). Time of the day showed a significant effect insofar as the subjective rating of affect decreased (energetic arousal) and increased (calmness) throughout the day. ICCs showed that 68/68/65% of the variance in the model was due to within-person and 32/32/35% due to between-person variance for valence/energetic arousal/calmness respectively.

PHYSICAL ACTIVITY FOLLOWING AFFECT

Between-person results of the predictors indicate no significant effect. However, results for the control variables showed that individuals with a higher BMI recorded significantly lower VM in the 15 minutes following the assessment. This results means that a person with a BMI value that is one point higher than the group average, recorded 11.03 less VM in the following 15 minutes (β=-0.06, *p*=0.027). In addition, the recorded VM of a person was significantly higher when the VM in the 15 minutes prior to the assessment was increased (β=0.26, *p*<0.001). ICCs showed that 86% of the variance in the model was due to within-person and 14% due to between-person variance.
