# Supplementary material for: Bi-directional associations of core affect and physical activity in adults with higher body weight: An ecological momentary assessment study
Source: J Health Psychol. 2024 Jan 29;29(10):1115–28. doi: 10.1177/13591053241228202 (PMC11344957; doi:10.1177/13591053241228202)
Supplement: sj-docx-3-hpq-10.1177_13591053241228202 – Supplemental material for Bi-directional associations of core affect and physical activity in adults with higher body weight: An ecological momentary assessment study [file sj-docx-3-hpq-10.1177_13591053241228202.docx]

STROBE Statement—checklist of items that should be included in reports of observational studies

|  | Item No. | Recommendation | Page  No. | Relevant text from manuscript |
| --- | --- | --- | --- | --- |
| **Title and abstract** | 1 | (*a*) Indicate the study’s design with a commonly used term in the title or the abstract | 1 (title page) | An Ecological Momentary Assessment Study |
|  |  | (*b*) Provide in the abstract an informative and balanced summary of what was done and what was found | 1 – 2 | “Dimensions of affect and levels of PA in free-living situations were assessed across 7 consecutive days via ecological momentary assessment (EMA) and accelerometry from 157 participants (68% female, BMI: 32.99±3.78 kg/m^2^).”  “Results suggest distinct acute bi-directional associations between dimensions of core affect and PA among individuals with overweight and obesity.” |
| Introduction | | | |  |
| Background/rationale | 2 | Explain the scientific background and rationale for the investigation being reported | 2 - 3 | “Individuals with obesity may develop implicit or explicit affective attitudes towards PA that arise from experienced weight discrimination, physical limitations and activity-related fears [8,9].“  “In individuals with a body mass index (BMI) within the normal weight range, EMA studies strengthen the reciprocal association between core affect and measures of PA in everyday life (e.g., step counts or raw measures like vector magnitude (VM)) over the course of a short time window (i.e., 10 and 15 minutes) or a whole day.”  “However, in the field of obesity research the number of EMA studies are limited [24], and published articles provide little knowledge about the within-person associations between core affect and PA.” |
| Objectives | 3 | State specific objectives, including any prespecified hypotheses | 3 - 4 | “Following previous findings on this topic, the current EMA study aimed to investigate the acute bi-directional association between estimates of PA (i.e., VM) and incidental affect (i.e, valence, energetic arousal, and calmness) in individuals with overweight and obesity at the within- and between-participant-level using accelerometer-derived PA counts continuously measured over seven consecutive days in everyday life.”  “The first objective was to examine whether PA in the 15 minutes prior to the EMA of affect is associated with participants reports of affect at the within-person levels. We hypothesized that higher levels of PA 15 minutes prior to an affect measurement occasion would be associated with higher reported levels of valence (H 1.1) as well as energetic arousal (H 1.2) and lower reported levels of calmness (H 1.3). The second objective was to investigate whether participants’ reports of affect would be associated with PA in the following 15 minutes at the within-person levels. We hypothesized that higher levels of valence (H 2.1) and energetic arousal (H 2.2) and lower levels of calmness (H 2.3) at one affect measurement occasion would be associated with higher levels of PA in the following 15 minutes. We further explored differences at the between-person level and controlled for both person-specific (sex, age, BMI) and study-related variables (day of the week, time of the day, day in the study).” |
| Methods | | | |  |
| Study design | 4 | Present key elements of study design early in the paper | 4 | “The present EMA study is a […]” |
| Setting | 5 | Describe the setting, locations, and relevant dates, including periods of recruitment, exposure, follow-up, and data collection | 4 - 5 | “Affect and PA was assessed from December 2019 to August 2020 as part of a baseline assessment.”  “Participants were recruited via social media, newspaper, radio, and self-help groups. Eligible individuals (see Additional file 1) were invited to a 1-hour appointment at the study sites in *details omitted for double-anonymized peer review* […]”  “The day after the in-person meeting, baseline measurements for PA and affect were assessed over seven consecutive days. Participants were informed about the group assignment after the baseline measurement was completed.”  “A mixed sampling scheme with both semi-random signals and participant-initiated prompts was applied. Participants chose their preferred time frame of assessment (6 a.m. – 9:30 p.m.; 7 a.m. – 10:30 p.m.; 8 a.m. – 11:30 p.m.; 9 a.m. – 12:30 a.m.; 10 a.m. – 1:30 a.m), which could be adapted during the one-week assessment. Participants were alerted by the app at random times within eight 90-minute block that were 30 minutes apart throughout the chosen time period.” |
| Participants | 6 | (*a*) *Cohort study*—Give the eligibility criteria, and the sources and methods of selection of participants. Describe methods of follow-up  *Case-control study*—Give the eligibility criteria, and the sources and methods of case ascertainment and control selection. Give the rationale for the choice of cases and controls  *Cross-sectional study*—Give the eligibility criteria, and the sources and methods of selection of participants | 5, 7, Additional File 2 | “For the current examination, only data from those participants who participated in the baseline assessment week and who both wore the accelerometer and answered at least one EMA questionnaire were included (n = 196, 68% female).”  “Participants that indicated an accelerometer wear-time of less than 10 hours for three or more days (non-valid days) were excluded. We further removed participants that completed less than 17 (30%) of the 56 EMA questionnaires (non-compliant) [20].”  “Ninty-three of the initial 196 participants were excluded from the analysis because they did not wear the sensor for at least 10h/day for at least four days (n = 4; 63 observations) or answered less than 17 EMA prompts (n = 35; 324 observations). The remaining 157 participants answered 6857 (M = 43.67, SD = 15.88, range: 17 – 98) EMA prompts while 8792 were planned to be delivered. Further, 2050 (15 min prior) and 2569 (15 min following) observations were excluded due to practical and statistical reasons, yielding a final observation count of 4807 (15 min prior) and 4276 (15 min following).” |
|  |  | (*b*) *Cohort study*—For matched studies, give matching criteria and number of exposed and unexposed  *Case-control study*—For matched studies, give matching criteria and the number of controls per case | *Not applicable* | *Not applicable* |
| Variables | 7 | Clearly define all outcomes, exposures, predictors, potential confounders, and effect modifiers. Give diagnostic criteria, if applicable | 5 | “Participants were instructed to respond to the prompt as soon as possible in everyday life. EMA questionnaires consisted of 18 items in total with an estimated response time between 30 and 120 seconds. Only the six bipolar items measuring affect were included in this examination. For these items, participants marked the point that represented their perception of their current state on a visual analogue scale. If participants missed a prompt or were not able to answer the questions at the moment, they were able to postpone the questions. Participants were not aware of the prompting schedule and were only informed that prompts would occur randomly in the chosen time span. Participants’ compliance with the sampling schedule was calculated from the completed number of assessments relative to the 56 scheduled assessments. In addition to the questionnaire, participants’ PA was measured continuously for seven days using an accelerometer.” |
| Data sources/ measurement | 8* | For each variable of interest, give sources of data and details of methods of assessment (measurement). Describe comparability of assessment methods if there is more than one group | 6 | “PA was measured continuously using the tri-axial ActiGraph® wGT3X-BT accelerometer (firmware v1.9.2, ActiGraph, Pensacola, FL, USA).”  “To assess incidental affect, the 6-item German short scale of the Multidimensional Mood Questionnaire [35] was used, which was developed and validated for momentary assessment [36].” |
| Bias | 9 | Describe any efforts to address potential sources of bias | *Not applicable* | *Not applicable* |
| Study size | 10 | Explain how the study size was arrived at | *Not applicable* | *Not applicable* |

Continued on next page

| Quantitative variables | 11 | Explain how quantitative variables were handled in the analyses. If applicable, describe which groupings were chosen and why | 6 - 7 | “Raw PA data was imported into the ActiLife® software (version 6.13.4; ActiGraph, Pensacola, FL, USA; see Additional File 1). PA data was matched with the EMA questionnaire responses using electronic date and time stamps within a Microsoft Access database. Following existing EMA studies that have investigated the proposed research questions within non-clinical settings, we included PA in the 15 minutes prior to and 15 minutes following the affect assessment [17, 18, 22]. Therefore, proceeding from the time stamps of the questionnaire (i.e., opening and completion), PA was calculated by aggregating the mean VM from 15 minutes prior to (i.e., 15 minutes before the questionnaire was opened) and 15 minutes following (starting from the time the questionnaire was completed) each completed EMA (Figure 1).” |
| --- | --- | --- | --- | --- |
| Statistical methods | 12 | (*a*) Describe all statistical methods, including those used to control for confounding | 7 – 8 | “Multilevel models were calculated with the repeated measurements (level 1) nested within participants (level 2) to identify within- and between-person effects. Four separate models were calculated to investigate the influence of PA in the 15 minutes prior to the EMA on affect (H 1.1 - 1.3) as well as the influence of affect on PA in the 15 minutes following the assessment (H 2.1 – 2.3).”  “All continuous predictors (PA in the 15 minutes prior, valence, calmness, energetic arousal), were centered at the person-mean by subtracting each individual’s score from their respective individual mean[39] and included into the respective models at level 1 as fixed effects. Random effects for each predictor were included in the model. Non-significant random effects were excluded, resulting in different models for the predictors. Next, we entered a series of variables at level 1 as fixed effects to control for timely and diurnal variations: weekday or weekend (weekday=0, weekend=1), time-of-day (dummy coded: imorning=00:00:00–11:59:59 (reference), afternoon=12:00:00–16:59:59, evening=17:00:00–23:59:59) and day in the study (0 – 6). PA in the 15 minutes prior to the prompt was added as an additional control variable at level 1 for the PA model. The person-mean of the respective predictor and sex (female=0, male=1), BMI (kg/m2, centered at grand-mean) and age in years (centered at grand-mean) were added as between-person variables at level 2 into the models (see Additional file 1).” |
|  |  | (*b*) Describe any methods used to examine subgroups and interactions | 8 | “The person-mean of the respective predictor and sex (female=0, male=1), BMI (kg/m2, centered at grand-mean) and age in years (centered at grand-mean) were added as between-person variables at level 2 into the models (see Additional file 1).” |
|  |  | (*c*) Explain how missing data were addressed | 7 | “Maximum likelihood estimations were used to estimate model parameters and guide the inclusion of control variables.” |
|  |  | (*d*) *Cohort study*—If applicable, explain how loss to follow-up was addressed  *Case-control study*—If applicable, explain how matching of cases and controls was addressed  *Cross-sectional study*—If applicable, describe analytical methods taking account of sampling strategy | *Not applicable* | *Not applicable* |
|  |  | (*e*) Describe any sensitivity analyses | *Not applicable* | *Not applicable* |
| Results | | | | |
| Participants | 13* | (a) Report numbers of individuals at each stage of study—eg numbers potentially eligible, examined for eligibility, confirmed eligible, included in the study, completing follow-up, and analysed | 7 (Figure 2), Additional File 2 | “Ninty-three of the initial 196 participants were excluded from the analysis because they did not wear the sensor for at least 10h/day for at least four days (n = 4; 63 observations) or answered less than 17 EMA prompts (n = 35; 324 observations). The remaining 157 participants answered 6857 (M = 43.67, SD = 15.88, range: 17 – 98) EMA prompts while 8792 were planned to be delivered. Further, 2050 (15 min prior) and 2569 (15 min following) observations were excluded due to practical and statistical reasons, yielding a final observation count of 4807 (15 min prior) and 4276 (15 min following).” |
|  |  | (b) Give reasons for non-participation at each stage | 7, Additional File 2 | “Participants that indicated an accelerometer wear-time of less than 10 hours for three or more days (non-valid days) were excluded. We further removed participants that completed less than 17 (30%) of the 56 EMA questionnaires (non-compliant) [20].” |
|  |  | (c) Consider use of a flow diagram | 7 (Figure 2) | Figure 2 |
| Descriptive data | 14* | (a) Give characteristics of study participants (eg demographic, clinical, social) and information on exposures and potential confounders | 8 (Table 1) | Table 1 |
|  |  | (b) Indicate number of participants with missing data for each variable of interest | *Not applicable* | Not applicable |
|  |  | (c) *Cohort study*—Summarise follow-up time (eg, average and total amount) | *Not applicable* | Not applicable |
| Outcome data | 15* | *Cohort study*—Report numbers of outcome events or summary measures over time | 8 (Table 1) | Table 1 |
|  |  | *Case-control study—*Report numbers in each exposure category, or summary measures of exposure |  |  |
|  |  | *Cross-sectional study—*Report numbers of outcome events or summary measures |  |  |
| Main results | 16 | (*a*) Give unadjusted estimates and, if applicable, confounder-adjusted estimates and their precision (eg, 95% confidence interval). Make clear which confounders were adjusted for and why they were included | 9 (Table 2, Table 3) | Table 2, Table 3 |
|  |  | (*b*) Report category boundaries when continuous variables were categorized | 8, 9 | “Next, we entered a series of variables at level 1 as fixed effects to control for timely and diurnal variations: weekday or weekend (weekday=0, weekend=1), time-of-day (dummy coded: imorning=00:00:00–11:59:59 (reference), afternoon=12:00:00–16:59:59, evening=17:00:00–23:59:59) and day in the study (0 – 6).”  “a 1-point increase in PA above the person-mean in the 15 minutes prior the assessment of affect was related to an average increase of 0.0027 higher energetic arousal (scale 0-100), and a decrease of -0.0012 calmness (scale 0-100)." |
|  |  | (*c*) If relevant, consider translating estimates of relative risk into absolute risk for a meaningful time period | *Not applicable* | Not applicable |

Continued on next page

| Other analyses | 17 | Report other analyses done—eg analyses of subgroups and interactions, and sensitivity analyses | 9, Additional File 2 | “Between-person results of the predictor showed a significant effect between PA and energetic arousal (β = 0.10, *p* = 0.014), indicating that participants with higher PA than the average person show higher values of energetic arousal.”  “Intraclass correlation coefficients (ICCs) of the null models indicated that 31% (valence), 33% (calmness), 25% (energetic arousal) and 7% (VM) were due to between-person differences.”  “The day of the week (week day, weekend) showed a significant effect on the ratings of affect. This results means that on a Saturday or Sunday participants rating of valence and calmness increased by 3.66 and 4.99 respectively. In addition, the rating of all three subscales of affect was significantly higher for male than for female participants (valence: β=0.11, p=0.02, energetic arousal: β=0.17, p<0.001, calmness: β=0.10, p=0.04) and significant age differences were found for valence and energetic arousal. Being one year older than the group average was associated with higher values (valence: β=0.10, p=0.028, energetic arousal: β=0.11, p=0.007). Time of the day showed a significant effect insofar as the subjective rating of affect decreased (energetic arousal) and increased (calmness) throughout the day. ICCs showed that 68/68/65% of the variance in the model was due to within-person and 32/32/35% due to between-person variance for valence/energetic arousal/calmness respectively. “  “Between-person results of the predictors indicate no significant effect. However, results for the control variables showed that individuals with a higher BMI recorded significantly lower VM in the 15 minutes following the assessment. This results means that a person with a BMI value that is one point higher than the group average, recorded 11.03 less VM in the following 15 minutes (β=-0.06, p=0.027). In addition, the recorded VM of a person was significantly higher when the VM in the 15 minutes prior to the assessment was increased (β=0.26, p<0.001). ICCs showed that 86% of the variance in the model was due to within-person and 14% due to between-person variance.” |
| --- | --- | --- | --- | --- |
| Discussion | | | | |
| Key results | 18 | Summarise key results with reference to study objectives | 10 – 12 | “The primary aim of the present EMA study was to investigate the bi-directional association between device-based measured PA and self-reported levels of valence, energetic arousal, and calmness in everyday life in a sample of individuals with overweight and obesity on the within-person level. The findings support the hypothesis of a bi-directional association between PA in daily life and energetic arousal (H 1.2 and H 2.2) and calmness (H 1.3 and H 2.3), whereas no evidence was found for valence (H 1.1 and H 2.1).”  “This null finding for valence (pleasant – unpleasant) is unexpected because from a theoretical perspective, positively valenced incidental affect is assumed to influence the cognition, perception and behavior of individuals insofar that beneficial resources are expanded and positive goals (i.e., health promotion) are pursued [40, 41].”  “As hypothesized, higher energetic arousal (energy - tiredness) was reported by the participants whenever they were more physically active in the 15 minutes prior to the assessment (H 1.2). The reverse analysis showed that higher energetic arousal was associated with being more physically active in the 15 minutes after the assessment (H 2.2).”  “We found evidence for a significant negative bi-directional association between PA and calmness as hypothesized. If individuals with overweight and obesity were more physically active, they felt more agitated (i.e., less calm and relaxed), and if participants felt calmer they were less physically active.” |
| Limitations | 19 | Discuss limitations of the study, taking into account sources of potential bias or imprecision. Discuss both direction and magnitude of any potential bias | 12 | “Nonetheless, some limitations must be considered when interpreting the results of this paper. First, PA data were not distinguished between exercise and non-exercise PA even though previous studies showed distinct associations to core affect [22]. Given the limited studies on participants with overweight and obesity, this study included only the raw measure of activity counts. Future studies should delve deeper and include other PA measures (i.e., light, moderate, and vigorous PA, step count) and contextual variables. Second, participants had unlimited time to respond to the prompts because the program had no timeout feature. Time was accounted for in the data processing, but it led to missing data. Including timeout for prompts could reduce participant burden in future research. Third, time windows of 15 minutes were chosen for the analysis of the bi-directional association of PA and affect according to previous literature [17,18]. Given the insufficient knowledge about the temporal course of these associations, various time windows should be explored in future studies [20].” |
| Interpretation | 20 | Give a cautious overall interpretation of results considering objectives, limitations, multiplicity of analyses, results from similar studies, and other relevant evidence | 10, 11, 12 | “An explanation for the deviating results could be the wide variety of study designs and assessments of affective valence used.” “Another reason could be that the positive effect of PA on valence and vice versa might hold over the course of the day in individuals with overweight and obesity but not immediately as shown in adults with normal weight [15, 45]. In studies using similar methods, our findings are comparable to the results of Kanning and colleagues [23] who also found that PA in the 10 minutes prior did not predict valence in older adults.”  “Based on our results, we assume that these divergent associations between PA and pleasantness are not only relevant for exercise (i.e., defined sports activities) but also prevalent in everyday movement-based behavior”  “This finding is reasonable because behavioral theories assume that positively valenced experiences are more likely to be repeated and therefore reinforce future PA behavior in everyday life [10]. However, the finding is particularly interesting in light of the fact that we detected no positive association between valence and PA. In our sample, higher levels of PA led to a high-activation (i.e., feeling awake and full of energy) but not more pleasantness.”  “It could be, for example, that higher levels of PA are associated with higher levels of stress [48] (e.g., running to catch a train ride) or internalized weight stigma [49] (e.g., walking up stairs while being watched by others). When developing PA interventions, researchers and practitioners should inform participants about the acute effects of PA (i.e., restlessness) and the long-term beneficial effects on well-being.” |
| Generalisability | 21 | Discuss the generalisability (external validity) of the study results | 13 - 14 | “Overall, the results suggest distinct acute bi-directional within-person associations between the dimensions of core affect and PA among adults with overweight and obesity in everyday life. Although more research is needed to confirm these findings, we suggest two important implications as a starting point for researchers and practitioners that might inform the development and design of (digital) interventions that target PA engagement.  First, the motivation to engage in long-term everyday PA among individuals with overweight and obesity could be enhanced by delivering health communication messages about the benefits of PA that go beyond the weight-related health outcomes. Psychoeducational material should be provided that highlights the affect-regulation potential of everyday PA. Moreover, our finding that being physically active led to no enhancement in feeling better contradicts the narrative often portrayed in PA programs (“move more and you will feel better”). Hence, this association might not apply as an acute effect to individuals with overweight and obesity and should thus be presented with caution. Such messages could establish a discrepancy between the expectations of PA and the actual experience of PA. Instead, the awareness of participants should be shifted to feelings of energy and agitation (i.e., psychoeducation and self monitoring after PA engagement), which occur even after short periods of PA and which might increase the adaption of a habitual PA lifestyle.  Second, our results suggest that feeling more energized and agitated predicted acute PA engagement in individuals with overweight and obesity. These findings could imply that interventions designed to foster PA might be more effective when they deliver brief affect-enhancing strategies that target the modification of energetic arousal and calmness in real-time. More specifically, individuals with overweight and obesity might benefit from guided mental imagery tasks about PA engagement or the confrontation with auditory or visual stimuli (i.e., high arousal rhythmic music, positive media content). In addition, situations in which an individual feels more energetic and less calm might represent an opportune moment for PA interventions (i.e., recommend PA when ratings of energetic arousal are high and ratings of calmness are low). The timing of such personalized acute interventions could be identified by affect focused assessments and the identification of individual patterns of affect fluctuation throughout a day and week (i.e., adaptive just-in-time interventions).  The replication of the present evidence on distinct influences of core affect and PA within other (more diverse) samples (i.e., BMI, gender, race, no treatment enrollment) is needed to generalize these findings and to confirm causality. Likewise, we encourage further studies to implement experimental based approaches (e.g., manipulation of energetic arousal) and to investigate factors that might moderate the nature of the reciprocal associations in individuals with overweight and obesity (e.g., internalized weight bias, physical limitations, context, intensity of PA, self-determination, self-efficacy, depression).” |
| Other information | |  | | |
| Funding | 22 | Give the source of funding and the role of the funders for the present study and, if applicable, for the original study on which the present article is based | 14 | [details omitted for double-anonymized peer review] |

*Give information separately for cases and controls in case-control studies and, if applicable, for exposed and unexposed groups in cohort and cross-sectional studies.

**Note:** An Explanation and Elaboration article discusses each checklist item and gives methodological background and published examples of transparent reporting. The STROBE checklist is best used in conjunction with this article (freely available on the Web sites of PLoS Medicine at http://www.plosmedicine.org/, Annals of Internal Medicine at http://www.annals.org/, and Epidemiology at http://www.epidem.com/). Information on the STROBE Initiative is available at www.strobe-statement.org.
